# Low serum HSPA12B levels are associated with an increased risk of sarcopenia in a Chinese population of older adults

**DOI:** 10.1016/j.cstres.2025.02.003

**Published:** 2025-02-19

**Authors:** Xin-Feng Jiao, Yue Gao, Ran Ni, Wen-Ya Zhao, Can Zhao, Xiang Lu, Hai-Feng Zhang, Wei Gao, Lan Luo

**Affiliations:** 1 Department of Geriatrics, Affiliated Hospital of Nantong University, Nantong, China; 2 Department of Geriatrics, Zhongda Hospital, School of Medicine, Southeast University, Nanjing, China; 4 Department of Geriatrics, The First Affiliated Hospital of Nanjing Medical University, Nanjing, China; 3 Department of Geriatrics, Sir Run Run Hospital, Nanjing Medical University, Nanjing, China

**Keywords:** HSPA12B, Sarcopenia, Older adults, Serum

## Abstract

Sarcopenia is a geriatric syndrome characterized by progressive loss of muscle mass and function. Heat shock protein (HSP) A12B is essential for angiogenesis and endothelial function. However, the association of HSPA12B levels with sarcopenia remains unclear. A total of 936 community-dwelling elderly people were recruited, and serum HSPA12B was measured by enzyme-linked immunosorbent assay. Appendicular skeletal muscle mass index (ASMI), grip strength, and gait speed were taken to assess sarcopenia. We found that serum HSPA12B levels in patients with sarcopenia (median [interquartile range] = 182.15 [137.58–225.86] ng/mL) were lower than those in elderly people without sarcopenia (228.96 [193.03–292.93] ng/mL, *P* < 0.001). Receiver operating characteristic curve analysis indicated that the optimal cut-off value of serum HSPA12B level for predicting sarcopenia was 185.50 ng/mL, with a sensitivity of 52.6% and a specificity of 80.8% (area under curve = 0.742, 95% confidence interval [CI] = 0.711–0.772, *P* < 0.001). Moreover, serum HSPA12B concentration was positively correlated with ASMI (*r* = 0.354, *P* < 0.001), grip strength (*r* = 0.381, *P* < 0.001), and gait speed (*r* = 0.169, *P* < 0.001). Multivariate logistic regression analysis showed that decreased serum HSPA12B levels (<185.50 ng/mL) were a risk factor for increased risk of sarcopenia (adjusted odds ratio = 4.335, 95% CI = 3.136–5.993, *P* < 0.001). In addition, serum HSPA12B level was also positively correlated with serum levels of angiogenesis markers, vascular endothelial growth factor (*r* = 0.080, *P* = 0.014), and angiopoietin-1 (*r* = 0.108, *P* = 0.001). In summary, our results indicate that low serum HSPA12B level is associated with an increased risk of sarcopenia in the elderly, suggesting a potential role of HSPA12B in the development of sarcopenia.

## Introduction

Sarcopenia is a geriatric syndrome characterized by low muscle mass and function.^1^, ^2^ The prevalence of sarcopenia is approximately 5–13% in people aged 60–70 years and 11–50% in people aged ≥80 years.^3^ In China, the prevalence of sarcopenia ranges from 9.8% to 18.6%.^4^, ^5^ Sarcopenia increases the risk of falls and fractures, reduces quality of life, and increases the risk of illness and even death. It has become a major challenge to the health of the elderly.^6^, ^7^ Diagnosing sarcopenia requires measuring muscle mass, muscle strength, and physical performance.^8^ However, due to differences in age, sex, disease status, degree of cooperation, cut-off values, and other factors, the accuracy and sensitivity of diagnosis remain suboptimal.^9^ Over the past few years, considerable efforts have been made to seek potential blood biomarkers to aid in the screening and diagnosis of sarcopenia; however, feasible noninvasive biomarkers for early identification of sarcopenia remain limited.

Heat shock protein (HSP) A12B is the newest member of the HSP70 family and is mainly cited in endothelial cells.^10^ Its presence is essential for angiogenesis and endothelial function in different species.^11^ HSPA12B gene therapy improved perfusion, promoted neovascularization, and reduced fibrosis in a mouse model of hindlimb ischemia.^12^ HSPs were reported to be present in plasma and serum, trigger innate immunity, and serve as disease biomarkers.^13^ For example, HSP70 was upregulated in the plasma of sarcopenia patients and may be a potential biomarker for the disease.^14^ Induction of muscle HSP70 improved insulin sensitivity and muscle performance in aged mice.^15^ However, it remains unclear whether serum HSPA12B levels are associated with the risk of sarcopenia. Therefore, we conducted a case-control study investigating the relationship between serum HSPA12B and sarcopenia in Chinese community-dwelling older adults.

## Results

### Baseline characteristics of the study participants

The study population included 936 older adults with 536 sarcopenia patients (Table 1). As expected, patients with sarcopenia had lower levels of appendicular skeletal muscle mass index (ASMI), grip strength, and gait speed compared to older adults without sarcopenia (*P* < 0.001). Patients with sarcopenia were older and had lower levels of body mass index (BMI), alanine transaminase, TBil, and triglyceride (TG) but higher levels of fasting blood glucose, total cholesterol, low-density lipoprotein cholesterol, and high-density lipoprotein cholesterol compared with the individuals without sarcopenia. Furthermore, a higher proportion of patients with sarcopenia suffered from diabetes compared to nonsarcopenic patients (*P* = 0.004). There were no significant differences between sarcopenic and nonsarcopenic patients regarding gender, smoking, drinking, hypertension, aspartate aminotransferase, and renal function. Significantly, serum HSPA12B levels were lower in patients with sarcopenia (median [interquartile range] = 182.15 [137.58–225.86] ng/mL) than those without sarcopenia (228.96 [193.03–292.93] ng/mL) (*P* < 0.001, Table 1).

**Table 1:** The characteristics of the enrolled subjects.

Variables	Nonsarcopenia (n = 400)	Sarcopenia (n = 536)	*P*
Age, years	76.0 (71.0–81.0)	77.0 (70.0–82.0)	0.041
Male, n (%)	243 (57.3)	293 (54.7)	0.431
BMI, kg/m^2^	25.19 (23.05–26.95)	22.36 (20.01–23.92)	<0.001
Smokers, n (%)	60 (15.0)	71 (13.2)	0.444
Drinkers, n (%)	45 (11.3)	50 (9.3)	0.336
Hypertension, n (%)	192 (48.0)	238 (44.4)	0.275
Diabetes, n (%)	50 (12.5)	105 (19.6)	0.004
FBG, mmol/L	5.47 (5.10–6.11)	5.63 (5.19–6.53)	0.003
ALT, U/L	16.18 (12.43–21.31)	15.12 (11.10–20.18)	0.006
AST, U/L	21.41 (19.00–26.00)	22.00 (18.53–27.00)	0.178
TBil, μmol/L	13.13 (10.95–16.90)	12.22 (9.50–15.88)	<0.001
SCr, μmol/L	71.83 (53.50–85.50)	66.53 (51.83–83.55)	0.093
BUN, mmol/L	5.63 (4.58–6.44)	5.47 (4.60–6.97)	0.092
TC, mmol/L	4.73 (4.13–5.39)	4.93 (4.27–5.48)	0.001
TG, mmol/L	1.41 (1.04–1.95)	1.12 (0.89–1.61)	<0.001
LDL-C, mmol/L	2.40 (1.88–2.78)	2.47 (1.88–3.08)	0.021
HDL-C, mmol/L	1.37 (1.21–1.56)	1.47 (1.24–1.74)	<0.001
HSPA12B, ng/mL	228.96 (193.03–292.93)	182.15 (137.58–225.86)	<0.001
Grip, kg	28.10 (22.50–33.90)	17.90 (15.53–24.88)	<0.001
Gait speed, m/s	1.05 (0.96–1.13)	0.96 (0.80–1.10)	<0.001
ASMI, kg/m^2^	7.10 (6.46–7.70)	5.62 (5.13–6.50)	<0.001

Abbreviations used: ALT, alanine transaminase; ASMI, appendicular skeletal muscle mass index; AST, aspartate aminotransferase; BMI, body mass index; BUN, blood urea nitrogen; FBG, fasting blood glucose; HDL-C, high-density lipoprotein cholesterol; LDL-C, low-density lipoprotein cholesterol; HSPA12B, heat shock protein A12B; Scr, serum creatinine; TBil, total bilirubin; TC, total cholesterol; TG, triglyceride.

### Association of serum HSPA12B with the risk of sarcopenia

**Fig. 1:**
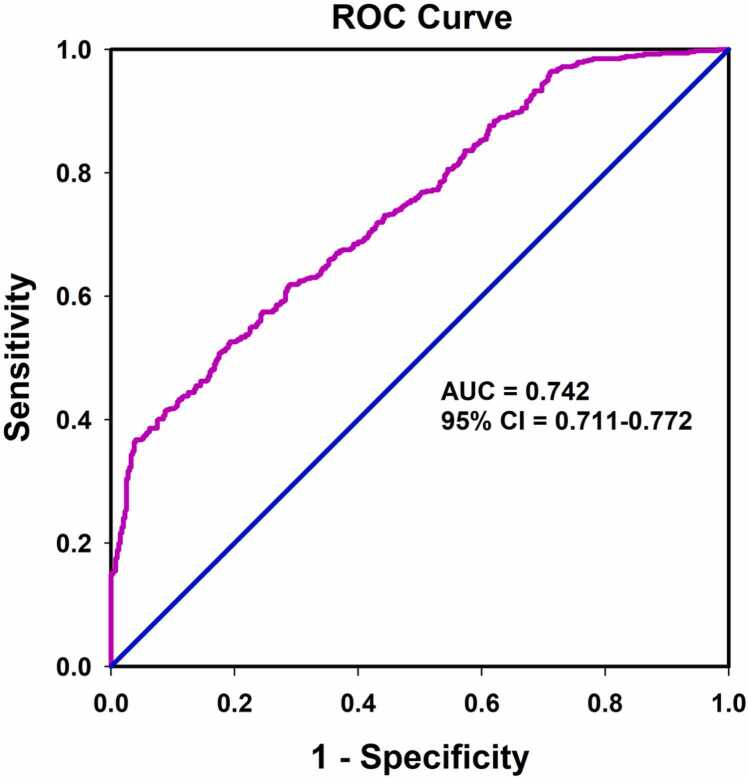
Receiver operating characteristic (ROC) curves for the diagnostic accuracy of Serum Metrnl for sarcopenia (n = 936). Abbreviation used: AUC, area under curve.

To analyze the association between HSPA12B and the risk of sarcopenia, we first inspected the correlation between HSPA12B and clinical variables. As shown in Table 2, serum HSPA12B levels were positively correlated with BMI, alanine transaminase, TBil, and TG but negatively correlated with high-density lipoprotein cholesterol. ROC curve analysis suggested that the optimal cut-off value for predicting sarcopenia was 185.50 ng/mL, with a sensitivity of 52.6% and a specificity of 80.8% (area under curve = 0.742, 95% confidence interval [CI] = 0.711–0.772, *P* < 0.001) (Figure 1). Univariate Logistic regression analyses showed that a range of variables such as BMI, diabetes mellitus, fasting blood glucose, TG, and serum HSPA12B levels may be associated with the risk of sarcopenia (Tables S1). Further multifactorial logistic regression analysis showed that low serum HSPA12B levels (<185.50 ng/mL) were significantly associated with an increased risk of sarcopenia (adjusted odds ratio [OR] = 4.335, 95% CI = 3.136–5.993, *P* < 0.001), even after correcting for the above potential confounders (Table 3). Similar results were observed when serum HSPA12B concentration was used as a continuous variable (adjusted OR = 1.015, 95% CI = 1.012–1.018, *P* < 0.001) (Table 3).

**Table 2:** Spearman’s correlation between serum HSPA12B and clinical variables.

Variables	HSPA12B	
(n = 936)	*r*	*P*
Age	−0.008	0.807
BMI	0.182	<0.001
FBG	−0.017	0.598
ALT	0.094	0.004
AST	0.040	0.221
TBil	0.107	0.001
Scr	0.015	0.694
BUN	−0.043	0.193
TC	−0.043	0.294
TG	0.068	0.036
LDL-C	−0.006	0.864
HDL-C	−0.084	0.010

Abbreviations used: ALT, alanine transaminase; ASMI, appendicular skeletal muscle mass index; AST, aspartate aminotransferase; BMI, body mass index; BUN, blood urea nitrogen; FBG, fasting blood glucose; HDL-C, high-density lipoprotein cholesterol; LDL-C, low-density lipoprotein cholesterol; HSPA12B, heat shock protein A12B; Scr, serum creatinine; TBil, total bilirubin; TC, total cholesterol; TG, triglyceride.

**Table 3:** Associations of serum HSPA12B with the risk of sarcopenia.

	Continuous		Categorical	
	OR (95% CI)	*P*	OR (95% CI)	*P*
Crude model	1.015 (1.012–1.017)	<0.001	4.024 (3.012–5.376)	<0.001
Adjusted model	1.015 (1.012–1.018)	<0.001	4.335 (3.136–5.993)	<0.001

n = 936. The adjusted model included body mass index, diabetes, fasting blood glucose, and triglyceride.

Abbreviations used: CI, confidence interval; HSPA12B, heat shock protein A12B; OR, odds ratio.

### Stratification analyses for the association of serum HSPA12B with the risk of sarcopenia

Stratified analyses were further conducted according to age, sex, diabetes, and BMI (Table 4). The association of low levels of HSPA12B with increased risk of sarcopenia remained significant both in older adults with the age of <80 years (adjusted OR = 5.003, 95% CI = 3.332–7.511, *P* < 0.001) and in those >80 years (adjusted OR = 3.168, 95% CI = 1.837–5.464, *P* < 0.001), as well as in males (adjusted OR = 4.072, 95% CI = 2.599–6.381, *P* < 0.001) and in females (adjusted OR = 4.505, 95% CI = 2.808–7.228, *P* < 0.001). Moreover, low levels of HSPA12B were also associated with increased risk of both in individuals with diabetes (adjusted OR = 4.004, 95% CI = 1.832–8.752, *P* < 0.001) and without diabetes (adjusted OR = 4.292, 95% CI = 3.004–6.132, *P* < 0.001), as well as in normal weight individuals (adjusted OR = 4.015, 95% CI = 2.590–6.224, *P* < 0.001) and in those overweight (adjusted OR = 4.389, 95% CI = 2.756–6.991, *P* < 0.001).

**Table 4:** Stratification analyses for the association of serum HSPA12B with the risk of sarcopenia.

	Continuous				Categorical			
Variables	Crude OR (95% CI)	*P*	Adjusted OR (95% CI)	*P*	Crude OR (95% CI)	*P*	Adjusted OR (95% CI)	*P*
Age								
<80	1.015 (1.012–1.018)	<0.001	1.016^a^(1.013–1.020)	<0.001	4.332 (3.033–6.186)	<0.001	5.003^a^(3.332–7.511)	<0.001
≥80	1.013 (1.009–1.018)	<0.001	1.013^a^(1.008–1.018)	<0.001	3.458 (2.098–5.698)	<0.001	3.168^a^(1.837–5.464)	<0.001
Sex								
Male	1.013 (1.010–1.016)	<0.001	1.013^a^(1.010–1.017)	<0.001	3.875 (2.614–5.745)	<0.001	4.072^a^(2.599–6.381)	<0.001
Female	1.019 (1.014–1.023)	<0.001	1.021^a^(1.015–1.026)	<0.001	4.203 (2.735–6.459)	<0.001	4.505^a^(2.808–7.228)	<0.001
Diabetes								
With	1.016 (1.009–1.022)	<0.001	1.015^b^(1.008–1.021)	<0.001	3.465 (1.675–7.168)	0.001	4.004^b^(1.832–8.752)	0.001
Without	1.015 (1.012–1.017)	<0.001	1.015^b^(1.012–1.018)	<0.001	4.079 (2.971–5.599)	<0.001	4.292^b^(3.004–6.132)	<0.001
BMI								
<24.0	1.015 (1.011–1.019)	<0.001	1.016^c^(1.012–1.019)	<0.001	3.746 (2.455–5.714)	<0.001	4.015^c^(2.590–6.224)	<0.001
≥24.0	1.014 (1.010–1.019)	<0.001	1.014^c^(1.010–1.018)	<0.001	4.671 (2.949–7.397)	<0.001	4.389^c^(2.756–6.991)	<0.001

Abbreviations used: BMI, body mass index; BUN, blood urea nitrogen; CI, confidence interval; FBG, fasting blood glucose; HDL-C, high-density lipoprotein cholesterol; hs-CRP, hypersensitive C-reactive protein; HSPA12B, heat shock protein A12B; OR, odds ratio; TG, triglyceride.

a The adjusted model included body mass index, diabetes, fasting blood glucose, and triglyceride.

b The adjusted model included body mass index, fasting blood glucose, and triglyceride.

c The adjusted model included diabetes, fasting blood glucose, and triglyceride.

### Association of serum HSPA12B with the severity of sarcopenia

**Fig. 2:**
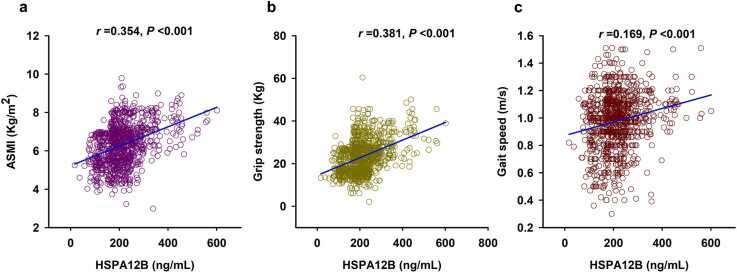
Correlation of serum HSPA12B level with ASMI (a), grip strength (b), and gait speed (c) (n = 936). Abbreviation used: ASMI, appendicular skeletal muscle mass index.

Severe sarcopenia is defined as low muscle strength, low muscle mass, and poor physical performance. We found that serum HSPA12B levels were positively correlated with ASMI, grip strength, and gait speed (Figure 2(a)-(c)). However, we did not find any significant difference in serum HSPA12B levels between patients with moderate sarcopenia and severe sarcopenia (181.80 [135.74–229.89] ng/mL vs. 183.03 [143.83–223.81] ng/mL, *P* = 0.511) (Figure S1).

### Association of serum HSPA12B with serum VEGF and Ang-1

**Fig. 3:**
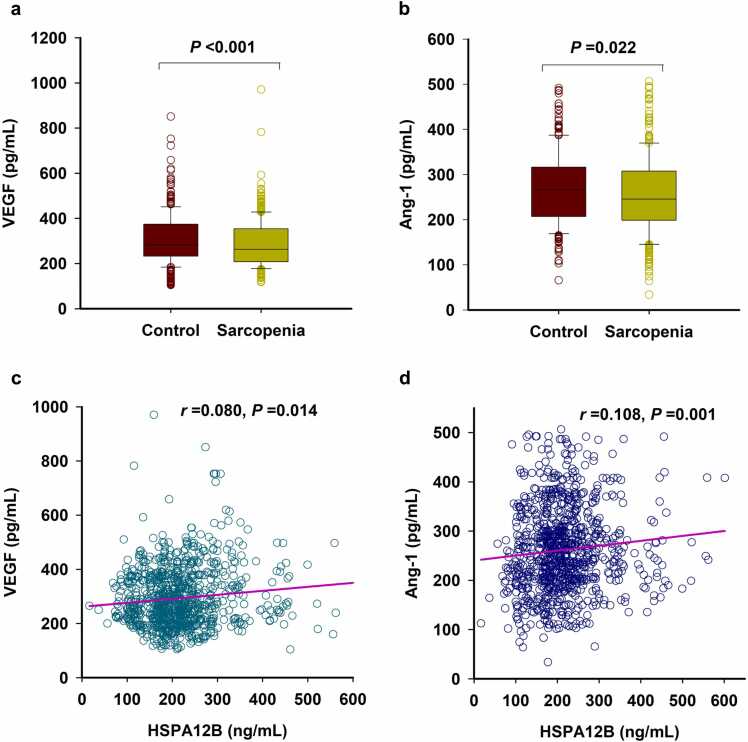
Association of serum HSPA12B level with serum VEGF and Ang-1 (n = 936). (a) and (b) Serum levels of VEGF (a) and Ang-1 (b). (c) and (d) Correlation of serum HSPA12B level with VEGF (c) and Ang-1 (d). Abbreviation used: VEGF, vascular endothelial growth factor.

We further examined the serum levels of two markers of angiogenesis, VEGF and Ang-1. As shown in Figure 3, serum VEGF (263.29 [207.82–353.33] pg/mL vs. 280.80 [232.93–373.76] pg/mL, *P* < 0.001) and Ang-1 (245.61 [198.85–307.42] pg/mL vs. 88.62 [77.80–106.01] pg/mL, *P* = 0.022) levels were both lower in patients with sarcopenia when compared to those control subjects (Figure 3(a) and (b)). Moreover, we also found that serum HSPA12B levels were positively correlated with serum levels of VEGF and Ang-1 (Figure 3(c) and (d)).

## Discussion

Our present study showed for the first time that serum HSPA12B, a protein with protective effects on angiogenesis and endothelial function, was associated with sarcopenia in older adults. Among 936 community-dwelling older adults, serum HSPA12B level was positively correlated with the components of sarcopenia, including skeletal muscle mass, grip strength, and gait speed. Low serum HSPA12B level (<185.50 ng/mL) was associated with an increased risk of sarcopenia in older adults.

The stress-induced 70 kDa heat shock protein (HSP70) is a highly conserved protein with multiple intracellular and extracellular functions.^19^ In skeletal muscle, HSP70 is rapidly induced in response to both noninjurious and injurious stress stimuli, including exercise and acute muscle injury.^20^, ^21^, ^22^ Up-regulation of HSP70 contributes to maintaining muscle fiber integrity and promotes muscle regeneration and recovery. In contrast, HSP70 expression is reduced during muscle inactivity and aging,^23^, ^24^, ^25^ and there is evidence to support that HSP70 deficiency may be a key mechanism contributing to muscle atrophy and contraction.^22^ Plasma HSPA12B was elevated in both septic mice and patients, indicating that HSPA12B may be a good predictor of poor prognosis in patients with severe sepsis.^26^ However, we showed here that serum HSPA12B levels were lower in patients with sarcopenia than nonsarcopenic subjects. Furthermore, low serum HSPA12B levels were positively correlated with decreased muscle mass, grip strength, and gait speed, suggesting a predictive role for HSPA12B concentrations in muscle dysfunction. In contrast, higher plasma HSP72 is associated with lower muscle mass, weaker grip strength, and slower walking speed, and has been considered a potential biomarker of sarcopenia in older adults.^14^ Sixteen weeks of resistance training decreased plasma HSP72 and increased muscle mass in sarcopenic men.^27^ However, more prospective studies with larger sample sizes from different regions are required to confirm the predictive role of circulating HSPA12B levels on the risk of sarcopenia.

Muscle weakness due to sarcopenia in the elderly is largely due to decreased capillary density in muscles, which is determined by local levels of various angiogenic factors that also decline in muscles with aging.^28^ Unlike most of the commonly expressed HSP70, HSPA12B was predominantly expressed in endothelial cells and required for angiogenesis.^11^ HSPA12B gene therapy improved perfusion, promoted neovascularization, and decreased fibrosis in a murine model of hindlimb ischemia.^12^ Meanwhile, HSPA12B overexpression protected the heart and brain from ischemic injury^29^, ^30^, ^31^, ^32^ as well as attenuated cardiac damage during endotoxemia.^33^ Conversely, endothelial HSPA12B knockout exacerbated sepsis-induced cardiac dysfunction.^34^ These effects are mediated by regulating the expression of proangiogenic factors such as Cox-2, VEGF, Ang-1, and eNOS.^29^, ^30^, ^31^, ^32^, ^33^, ^34^ Consistently, our results showed that serum levels of VEGF and Ang-1 were both lower in patients with sarcopenia. Moreover, serum HSPA12B level was also positively correlated with the levels of VEGF and Ang-1. Therefore, our findings indicate that HSPA12B may prevent the process of skeletal sarcopenia by promoting neovascularization. Furthermore, inflammation has been identified as one of the key hallmarks associated with aging and aging-related diseases including sarcopenia. Endothelial HSPA12B can inhibit inflammatory response under a variety of stress conditions, including lipopolysaccharide-induced sepsis,^35^ neuroinflammation,^36^ and sepsis-induced cardiomyopathy.^34^, ^37^ These findings suggest that HSPA12B may play a key role in suppressing inflammatory responses. However, the mechanism of the protective effect of HSPA12B on the development of skeletal sarcopenia requires further in-depth studies.

Our stratified analyses showed that the correlation between low HSPA12B levels and the risk of sarcopenia was prominent among participants over and under 80 years old. Serum HSP70 concentrations were significantly lower in community-dwelling elderly individuals than in healthy young control subjects.^38^ In contrast, muscle HSP70 levels were higher in older people compared to the young.^39^ Moreover, our stratified analyses revealed low levels of HSPA12B were also associated with increased risk both in individuals with diabetes and without diabetes. Serum HSP70 levels were significantly increased in diabetic patients compared with controls and correlated with disease duration.^40^ On the contrary, reduced expression of plasma HSP70 may be relevant to obesity and type 2 diabetes, and HSP70 concentration was negatively correlated with insulin resistance.^41^ In addition, higher levels of skeletal muscle HSP70 prevented the development of insulin resistance during healthy aging.^42^ Heterogeneity in sample types, study populations, and methodological designs may contribute to the inconsistency of these studies. However, the exact correlation between HSPA12B levels and the risk of sarcopenia in older adults with different states still needs to be further demonstrated.

### Study limitations

Although we found for the first time that low serum HSPA12B levels are associated with an increased risk of sarcopenia in the elderly, our current study should be interpreted in the context of certain limitations. First, our study subjects were from the Chinese population only and could not represent global regional levels. Second, although our study population was from both rural and urban areas, the cross-sectional nature made it difficult to rule out the possibility of selection and causal bias. Therefore, further prospective cohort studies are needed to confirm our findings. Third, although our results showed that serum HSPA12B was positively correlated with the components of sarcopenia; however, the correlation coefficients were relatively weak. Moreover, the area under curve of ROC curve was only 0.742, with a sensitivity of 52.6% and a specificity of 80.8%, indicating a relatively low predictive value. Indeed, by using 185.50 ng/mL as cut-off value, the false positive rate and the false negative rate are 21.4% and 44.0%, respectively. These results reveal that serum HSPA12B may not be a perfect biomarker for the diagnosis of sarcopenia. Finally, we did not test the correlation between serum HSPA12B levels and inflammatory factors to test the hypothesis that low HSPA12B concentrations may represent a chronic inflammatory condition.

## Conclusion

In conclusion, our findings suggest that lower serum HSPA12B levels are relevant to the risk of sarcopenia in older adults. Further studies on this chaperone protein may help to shed light on the complex pathogenesis of sarcopenia.

## Materials and methods

### Study participants

A total of 936 older adults aged ≥65 years were recruited from both rural and urban areas of Jiangsu Province, as previously described.^16^ Participants with the following conditions were excluded, including loss of independent mobility, inability to complete the required movements due to systemic chronic diseases, severe hepatic damage and renal failure, and malignant tumors. This study was conducted under the principles outlined in the Declaration of Helsinki^17^ and approved by the Ethics Committee of Sir Run Run Hospital, Nanjing Medical University (approval number 2019-SR-S041). Written informed consent was signed by all participants.

### Data collection

After overnight fasting, venous blood was collected in the early morning and separated into serum and cell components within 2 h. All samples with hemolysis or coagulation were discarded. The serum was stored at −80 °C for further analysis. A series of blood biochemical indicators were measured, as mentioned previously.^16^ Participants who smoked more than one cigarette per day in the past 12 months were classified as current smokers. Current drinkers were those who drank at least once a day in the past 12 months.^18^

### Assessment of sarcopenia

Sarcopenia was diagnosed based on the latest criteria of the Asian Working Group on Sarcopenia in 2019.^1^ Briefly, elderly patients with low muscle mass (ASMI <7.0 kg/m^2^ in men and <5.7 kg/m^2^ in women) and low muscle strength (grip strength <28 kg in men and <18 kg in women) and/or low physical function (walking speed <1.0 m/s) were categorized as having sarcopenia.^1^ Patients with low muscle mass combined with low muscle strength or low physical performance were assumed to have moderate skeletal sarcopenia. Patients with low muscle mass, low muscle strength, and low physical performance were assumed to have severe skeletal sarcopenia.^1^

### Serum HSPA12B, VEGF, Ang-1 measurements

According to the manufacturer's protocol, serum levels of HSPA12B (Cat. No. JLC6292, JINGKANG, China), vascular endothelial growth factor (VEGF) (Cat. No. JLC7465, JINGKANG, China), and Angiopoietin-1 (Ang-1) (Cat. No. JLC7486, JINGKANG, China) were measured by using enzyme-linked immunosorbent assay kits.

### Statistical analysis

The Kolmogorov-Smirnov test was used to test the normality of continuous variables, described as the median (interquartile range). The Mann-Whitney U test was utilized to determine differences between the two groups. The Pearson χ^2^ test was used to compare qualitative variables expressed as frequencies. Receiver operating characteristic (ROC) curve analysis was performed to determine the optimal cut-off value of serum HSPA12B levels for the best prediction of sarcopenia. Spearman's correlation was used to calculate the correlation between clinical variables. Univariate and multivariate logistic regression analyses were used to identify variables contributing to the presence of sarcopenia. Odds ratios (ORs) and 95% CIs were calculated. All tests were two-sided, and *P* < 0.05 was considered statistically significant. All analyses were performed using SPSS 28.0 (IBM, Chicago, IL).

## Ethics statement

This study was performed in accordance with the principles outlined in the Declaration of Helsinki and approved by the Ethics Committee of Sir Run Run Hospital, Nanjing Medical University (2019-SR-S041). Written informed consent was obtained from each participant.

## Funding and support

This work was supported by grants from the National Key Research and Development Plan of China (No. 2020YFC2008505 to Xiang Lu), the National Natural Science Foundation of China (No. 81970217 to Wei Gao), the project of Zhongda Hospital Affiliated to Southeast University for cultivating academic talent (No. CZXM-GSPRC22 to Wei Gao), the Zhongda Hospital Affiliated to Southeast University, Jiangsu Province High-Level Hospital Pairing Assistance Construction Funds (No. ZDLYG10 to Wei Gao), the Zhongda Hospital Affiliated to Southeast University, Jiangsu Province High-Level Hospital Construction Funds (No. GSP-LCYJFH17 to Wei Gao), the Jiangsu Commission of Health (No. LKZ2023005 to Wei Gao), the Jiangsu Provincial Research Hospital (No. YJXYY202204-XKA03 to Lan Luo), the Scientific research project from Jiangsu Health Commission (No. Ym2023113 to Lan Luo), and the Medical Project from Jiangsu Commission of Health (No. M2020033 to Lan Luo).

## Author contributions

W.G., H.F.Z., and L.L. contributed to the conception and design of the study. X.F.J., Y.G., C.Z., and W.Y.Z. contributed to data acquisition. W.G., X.L., and R.N. analyzed the data. X.F.J., Y.G., and R.N. drafted the manuscript. W.G. and H.F.Z. revised the manuscript. All authors read and approved the final submission.

## Consent for publication

Not applicable.

## Clinical trial number

Not applicable.

## CRediT authorship contribution statement

**Jiao Xin-Feng:** Writing – original draft, Methodology, Investigation, Formal analysis, Data curation. **Luo Lan:** Writing – review & editing, Validation, Funding acquisition, Conceptualization. **Gao Wei:** Writing – review & editing, Validation, Supervision, Funding acquisition, Conceptualization. **Zhang Hai-Feng:** Writing – review & editing, Validation, Supervision, Conceptualization. **Lu Xiang:** Validation, Supervision, Funding acquisition. **Zhao Can:** Supervision, Resources, Investigation. **Zhao Wen-Ya:** Resources, Investigation, Formal analysis. **Ni Ran:** Writing – original draft, Validation, Investigation, Formal analysis. **Gao Yue:** Writing – original draft, Methodology, Investigation, Formal analysis.

## Declarations of interest

The authors declare that there is no conflict of interest regarding the publication of this article.

## Data Availability

Data will be made available on request.
