# Supplementary material for: Low serum HSPA12B levels are associated with an increased risk of sarcopenia in a Chinese population of older adults
Source: Cell Stress Chaperones. 2025 Feb 19;30(2):100–8. doi: 10.1016/j.cstres.2025.02.003 (PMC11909431; doi:10.1016/j.cstres.2025.02.003)
Supplement: Supplementary file 1 — Supplementary material [file mmc1.docx]

**Table S1. Univariate analysis for the associations of clinical variables with the risk of sarcopenia.**

| **Variables** | **OR (95% CI)** | ***P*** |
| --- | --- | --- |
| **Age** | 0.995 (0.972-1.018) | 0.650 |
| **Male** | 0.942 (0.656-1.351) | 0.744 |
| **BMI** | 0.738 (0.699-0.780) | <0.001 |
| **Smokers**  **Drinkers**  **Hypertension**  **Diabetes** | 1.227 (0.757-1.989)  1.043 (0.596-1.824)  0.958 (0.686-1.339)  1.681 (1.051-2.688) | 0.405  0.883  0.803  0.030 |
| **FBG** | 1.182 (1.066-1.311) | 0.001 |
| **ALT** | 0.981 (0.960-1.003) | 0.086 |
| **AST** | 1.013 (0.990-1.035) | 0.273 |
| **TBil** | 0.994 (0.970-1.019) | 0.645 |
| **SCr** | 1.006 (0.998-1.013) | 0.134 |
| **BUN** | 1.006 (0.911-1.110) | 0.911 |
| **TC** | 1.084 (0.855-1.374) | 0.505 |
| **TG** | 0.728 (0.580-0.914) | 0.006 |
| **LDL-C** | 1.182 (0.877-1.594) | 0.272 |
| **HDL-C** | 1.389 (0.790-2.445) | 0.254 |
| **HSPA12B** | 1.015 (1.012-1.017) | <0.001 |

ALT, alanine transaminase; AST, aspartate aminotransferase; BMI, body mass index; BUN, blood urea nitrogen; CI, confidence interval; FBG, fasting blood glucose; HDL-C, high-density lipoprotein cholesterol; HSPA12B, Heat shock protein A12B; LDL-C, low-density lipoprotein cholesterol; OR, odds ratio; Scr, serum creatinine; TBil, total bilirubin; TC, total cholesterol; TG, triglyceride.
