# Supplementary figures and images for: Low serum HSPA12B levels are associated with an increased risk of sarcopenia in a Chinese population of older adults
Source: Cell Stress Chaperones. 2025 Feb 19;30(2):100–8. doi: 10.1016/j.cstres.2025.02.003 (PMC11909431; doi:10.1016/j.cstres.2025.02.003)

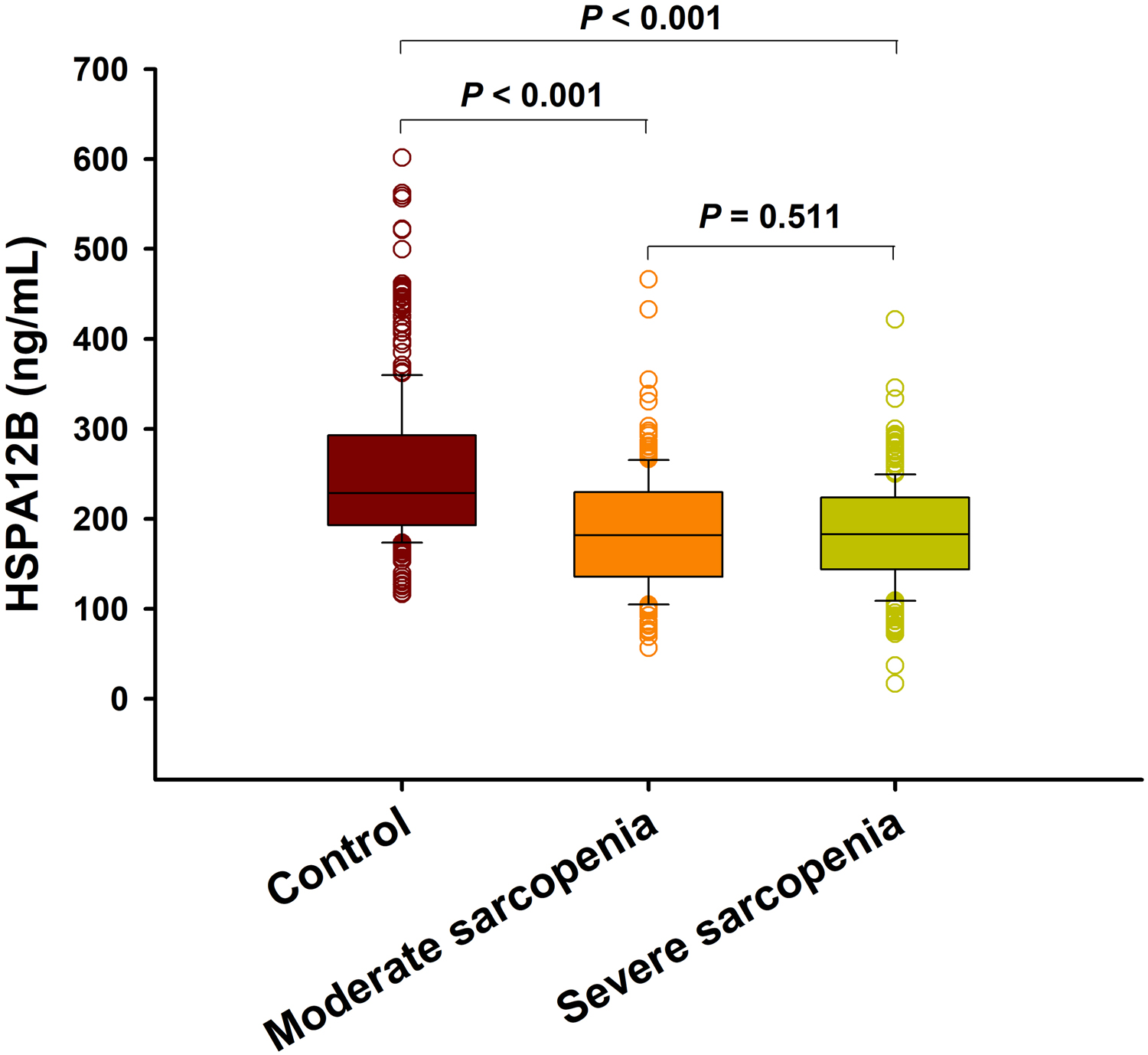

Supplement: Supplementary file 2 — Supplementary material [file mmc2.jpg]
